# 2-Deoxyglucose-Modified Folate Derivative: Self-Assembling Nanoparticle Able to Load Cisplatin

**DOI:** 10.3390/molecules24061084

**Published:** 2019-03-19

**Authors:** Shaoming Jin, Zhongyao Du, Pengjie Wang, Huiyuan Guo, Hao Zhang, Xingen Lei, Fazheng Ren

**Affiliations:** 1 Beijing Advanced Innovation Center for Food Nutrition and Human Health, College of Food Science & Nutritional Engineering, China Agricultural University, Beijing 100083, China; myjackyming@126.com (S.J.); duzhongyao@cau.edu.cn (Z.D.); wpj1019@cau.edu.cn (P.W.); XL20@cornell.edu (X.L.); 2 National Institutes for Food and Drug Control, Beijing 100050, China; 3 Beijing Laboratory of Food Quality and Safety, Beijing Higher Institution Engineering Research Center of Animal Product, Beijing 100083, China; guohuiyuan99@gmail.com (H.G.); zhanghaocau@cau.edu.cn (H.Z.); 4 Department of Animal Science, Cornell University, Ithaca, NY 14853, USA

**Keywords:** folic acid, 2-Deoxyglucose, drug delivery system, cisplatin, self-assembly

## Abstract

Folic acid has been widely introduced into nano-drug delivery systems to give nanoparticle-targeted characteristics. However, the poor water solubility of folic acid may hinder the exploitation of its ability to load antineoplastic drugs. In the present study, we designed a new folate derivative (FA-2-DG) synthesized from folic acid and 2-Deoxyglucose (2-DG). The aim of this study was to evaluate the self-assembly characteristics of FA-2-DG, and its ability of loading cisplatin. The critical micelle concentration was 7.94 × 10^−6^ mol L^−1^. Fourier transform infrared spectroscopy indicated that hydrogen bonding interaction is a main driving force for the self–assembly of FA-2-DG. The particle was stable in pure water or 0.5% bovine serum albumin dispersions. By forming a coordination bond, the particles assembled from FA-2-DG can load cisplatin. The loading efficiency was maximal when the molar ratio of FA-2-DG to cisplatin was 2:1.

## 1. Introduction

The targeting of active substances to the tumor region is an effective chemotherapy therapeutic protocol for cancer. Folic acid has carcinoma-associated effects due to the high expression tumor cell membrane folate receptors (FRs) [1,2,3,4]. FR α and FR β are two forms of FRs, which have high binding affinity with folic acid. Most normal tissues lack expression of FRs, making selective tumor targeting on the basis of folic acid achievable [5].

The superiority of the targeted nanoparticle drug delivery system makes it widely attractive in anticancer therapy research. Folic acid has been widely introduced to make nanoparticles with targeted characteristics [6,7,8]. Although known as a water-soluble vitamin, the water solubility of folic acid is poor and greatly affected by the pH value of the surrounding chemical environment, which may hinder the development of its loading ability for antineoplastic drugs. To overcome this problem, many forms of folic-acid-based nanoparticles have been developed [9,10,11,12,13,14,15,16,17,18,19]. One of the most effective ways to improve the water solubility of folic-acid-based nanoparticles is glycosylation modification [20,21,22,23]. 

2-Deoxyglucose (2-DG) is a structural analogue of glucose. In addition to having good water solubility similar to glucose, it also has biological functions, such as inhibiting glycolysis and reversing cancer cell metastasis [24,25,26,27]. These characteristics make 2-DG a good candidate for improving water solubility in antineoplastic therapy.

Cisplatin, a first-line clinical antitumor agent, has been widely used in the treatment of many kinds of malignant tumors, such as breast cancer, ovarian cancer, and lung cancer [28,29,30]. Its better curative effect also brings greater toxicity, and it takes up to 20 days after each administration to re-administer. Therefore, in order to reduce toxicity, research about cisplatin mostly uses the drug-loading system to target cisplatin to the tumor site [31,32]. 

**Figure 1:**
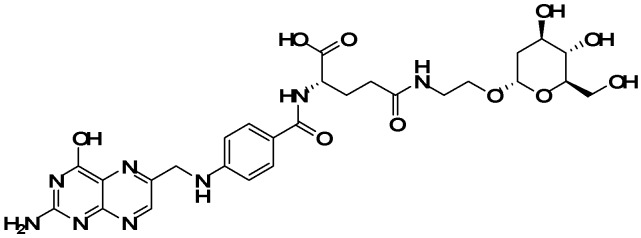
Chemical structure of FA-2-DG.

In the present study, we designed a new folate derivative (FA-2-DG) synthesized from folic acid and 2-DG, with amino ethanol as a linker agent, and the structure is shown in Figure 1. The aim of the study was to evaluate the self-assembly characteristics of FA-2-DG and its ability of loading cisplatin. 

## 2. Results

### 2.1. Water Solubility of FA-2-DG

**Figure 2:**
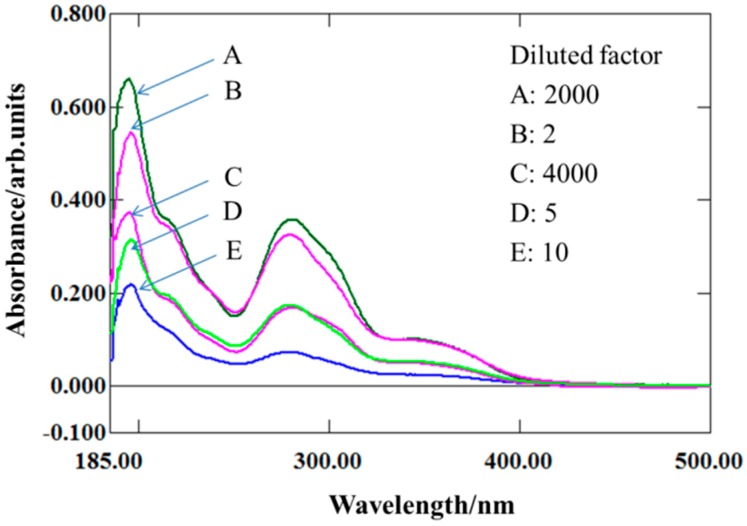
UV-VIS absorption spectra of different dilutions of saturated folic acid or saturated FA-2-DG solutions. (**A**) 2000-fold dilution of saturated FA-2-DG solution. (**B**) 2-fold dilution of saturated folic acid solution. (**C**) 4000-fold dilution of saturated FA-2-DG solution. (**D**) 5-fold dilution of saturated folic acid solution. (**E**) 10-fold dilution of saturated folic acid solution.

To evaluate the enhancement of water solubility by the introduction of 2-DG, the UV-VIS absorption spectra of a series of diluted FA-2-DG saturated solutions and folic acid solution in pure water were determined. The saturated folic acid solution was diluted 2-, 5-, and 10-fold, corresponding to B, D, and E in Figure 2, respectively. The saturated FA-2-DG solution was diluted 2000- and 4000-fold, corresponding to A and C in Figure 2, respectively. The results shown in Figure 2 indicate that the absorption at a wavelength of 280 nm of solution B is close to that of solution A, although a little lower, whereas the absorption of solution D at a wavelength of 280 nm is nearly the same as that of solution C. To summarize, the solubility of folic acid in aqueous solution increases by 800-fold with the introduction of 2-DG into its molecular structure. All the experiments were performed at a constant temperature of 37 °C to ensure the rigor of the test results.

### 2.2. Intermolecular Hydrogen Bonding

**Figure 3:**
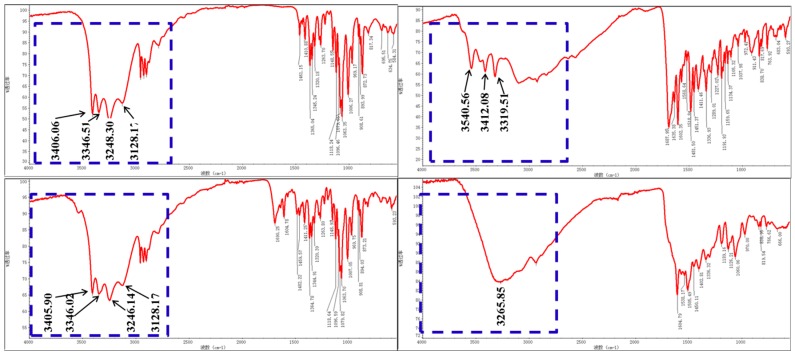
FTIR spectra of (**A**) 2-DG, the signals at 3406 cm^−1^, 3346.51 cm^−1^, 3248.30 cm^−1^, and 3128.17 cm^−1^ in blue dotted box represent the O–H peaks; (**B**) folic acid, the signals at 3540.56 cm^−1^, 3412.08 cm^−1^, and 3319.51 cm^−1^ represent the N–H and O–H peaks; (**C**) a physical mixture of folic acid and 2-DG (FA-2-DG/PM), the signals represented by their respective structures are also obvious; and (**D**) FA-2-DG, none of the N–H and O–H peaks can be clearly identified.

The FTIR spectra of different samples are shown in Figure 3. The blue dotted box marked in each spectrum outlines the locations of O–H and N–H signals, which are donors or receptors of intermolecular hydrogen bonds [33]. For 2-DG, the peaks of four O–H stretching vibrations occur at 3406 cm^−1^, 3346.51 cm^−1^, 3248.30 cm^−1^, and 3128.17 cm^−1^, as shown in Figure 3A. Figure 3B shows the FTIR spectrum of folic acid, in which N–H and O–H peaks occur at 3540.56 cm^−1^, 3412.08 cm^−1^, and 3319.51 cm^−1^. In the spectrum of a physical mixture of folic acid and 2-DG (FA-2-DG/PM), shown in Figure 3C, peaks representing O–H stretching vibrations are still present, indicating that there are no intermolecular hydrogen-bonding interactions between folic acid and 2-DG. However, in Figure 3D, none of the N–H and O–H peaks can be clearly identified, but are replaced by one broad band, which is a clear indication of strong hydrogen-bonding interactions between the molecules. This phenomenon indicates that the molecules can self-assemble under the appropriate conditions.

### 2.3. Critical Micelle Concentration (CMC) of FA-2-DG

**Figure 4:**
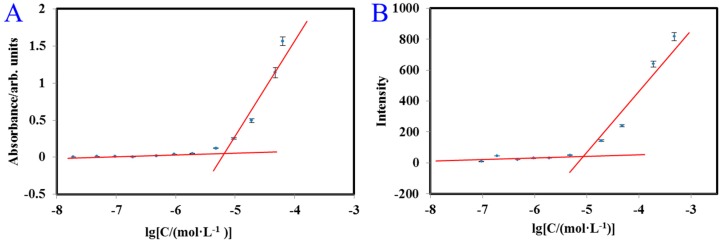
Determination of CMC of FA-2-DG by plotting of the UV absorbance at 280 nm (**A**) and fluorescence intensity at 442 nm (**B**) against the logarithm of the concentration.

A fluorescent probe, such as naphthalene, is often used to measure critical micelle concentration (CMC) [34]. However, FA-2-DG emits a fluorescent signal when excited at 280 nm, and its range of emission wavelengths overlaps that of naphthalene. Therefore, naphthalene was not applicable as a fluorescent probe in this study. As mentioned above, when the water solubility of FA-2-DG was determined from its UV-VIS absorption spectrum. Its maximum absorption wavelength was 280 nm, and it also emitted fluorescence at 442 nm. Therefore, we used UV-VIS and fluorescence spectroscopy to determine the CMC of FA-2-DG. As shown in Figure 4, the CMCs determined with UV-VIS and fluorescent spectra were nearly the same, and from the intersection of the two trend lines, we concluded that the CMC of FA-2-DG is 7.94 × 10^−6^ mol L^−1^.

### 2.4. Dynamic Light Scattering (DLS) Measurement of FA-2-DG

**Figure 5:**
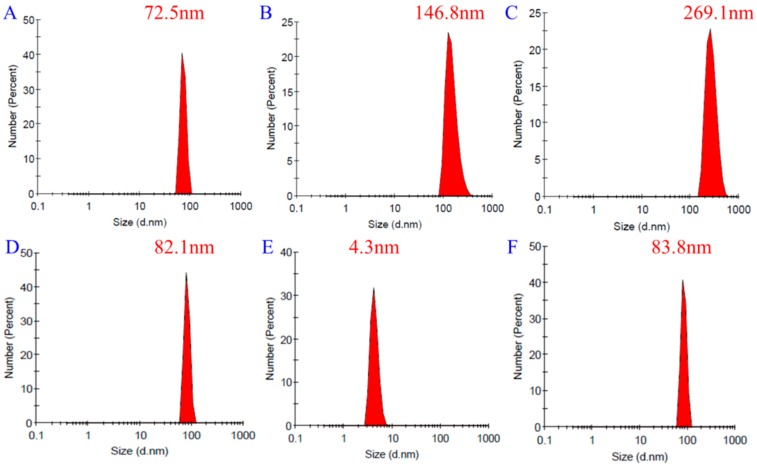
DLS results for FA-2-DG at concentrations of (**A**) 8 × 10^−6^ mol L^−1^ in pure water, (**B**) 14 × 10^−6^ mol L^−1^ in pure water, (**C**) 47 × 10^−6^ mol L^−1^ in pure water, (**D**) 9.5 × 10^−6^ mol L^−1^ in pure water, (**E**) 0.5% bovine serum albumin (BSA) aqueous solution without FA-2-DG, and (**F**) 9.5 × 10^−6^ mol L^−1^ in aqueous 0.5% BSA solution.

DLS measurements can provide the hydration diameter of particles in solution. Here, we determined the diameter of the particles in three FA-2-DG aqueous solutions of different concentrations. To confirm that the self-assembly of the molecules persisted in plasma, FA-2-DG was also dispersed in 0.5% aqueous bovine serum albumin (BSA) solution at a concentration of 9.5 × 10^−6^ mol L^−1^. From Figure 5A–C, we can see that the particle size increased as the concentration increased. To test the serum stability of the self-aggregated compound, we compared the particle size of FA-2-DG in BSA solution and in pure water. The results shown in Figure 5D–F indicate that the particle size was not influenced by BSA. Therefore, the aggregates were not disaggregated in BSA solution.

### 2.5. Ability of FA-2-DG to Load Cisplatin

**Figure 6:**
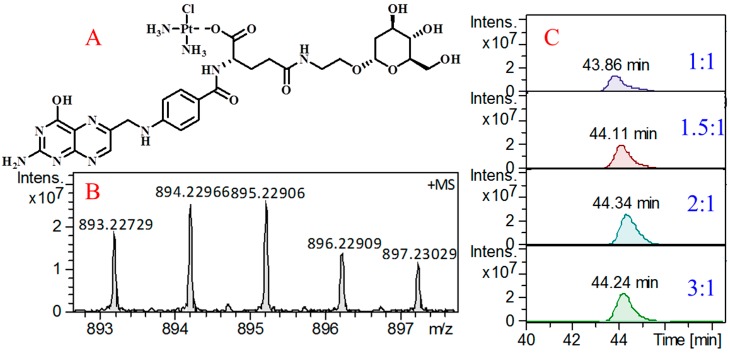
The structural characteristics of the compound. (**A**) Structure of the compound after the formation of the coordination bond; (**B**) Quasimolecular ion peaks of the precise molecular mass of this compound; (**C**) Extracted ion chromatogram at 895.22906 with different molar ratios.

The free carboxyl group in the structure of FA-2-DG can be used for drug loading [35]. Cisplatin, a first-line antineoplastic drug with weak water solubility, is often studied to improve its bioavailability, and many nanodrug-loading systems have been developed to achieve this goal [32,36,37]. These studies have shown that forming coordination bonds between carriers and cisplatin is a very effective strategy. FA-2-DG can form coordination bonds with cisplatin by substituting one chloride ion in the cisplatin molecule [38]. The structure shown in Figure 6A represents the compound after the formation of the coordination bond. To verify the correctness of this structure, the compound was analyzed with high-resolution mass spectrometry (MS). Figure 6B shows the quasimolecular ion peaks of the precise molecular mass of this compound. The excellent matching of the isotope peaks confirms the introduction of the platinum atom into the molecule.

To evaluate the loading efficiency of FA-2-DG for cisplatin, a set of solutions with different molar ratios of FA-2-DG and cisplatin were prepared and high-performance liquid chromatography (HPLC)–MS was used for the quantitation of the coordination compound. As shown in Figure 6C, we prepared four kinds of solutions with molar ratios of 1:1, 1.5:1, 2:1, and 3:1. The extracted ion chromatogram at 895.22906 corresponds, from top to bottom, to increasing proportions of FA-2-DG. The peak area was maximal at a molar ratio of 2:1, indicating the highest loading efficiency at this ratio. It also shows that the proportion of loaded molecule and the loading efficiency do not have a simple linear relationship, so that maximizing the loading efficiency will require individualized adjustment for different drugs.

## 3. Materials and Methods 

### 3.1. Preparation of FA-2-DG

The preparation of FA-2-DG has been reported previously [39]. The structure of FA-2-DG was confirmed with FTIR, nuclear magnetic resonance, and MS. All solvents and biochemical reagents were of analytical grade and were purchased from commercial sources (J&K Scientific, Beijing, China; Solarbio Science & Technology, Beijing, China).

### 3.2. Optical Characterization Method

UV-VIS spectroscopy (UV-2600, Shimadzu, Kyoto, Japan) was used to determine the absorption of FA-2-DG and folic acid in samples at different concentrations [40]. All spectra were measured at 37 °C with a quartz cuvette with a 1 cm path length, and recorded in a scan range of 220–800 nm.

Fluorescence emission spectroscopy (F-4500, Hitachi, Tokyo, Japan) was used to detect the emission intensities of the different samples [41]. Sample preparation was the same as for the UV-VIS analysis. The samples were excited at 280 nm and an emission spectrum was recorded from 300 to 600 nm. All of the fluorescence measurements were made at 37 °C.

The FTIR analysis (Nicolet iS5, Thermo Fisher Scientific, Waltham, MA, USA) was performed with the attenuated total reflectance method [42]. The samples, in powdered form, were analyzed in an IR frequency range of 500–4000 cm^−1^.

### 3.3. DLS Characterization

A DLS instrument (Zetasizer Nano ZS, Malvern, UK) was used to measure the particle size of FA-2-DG at different concentrations and in different solvents [43]. All particle diameter measurements were made at 37 °C.

### 3.4. Cisplatin Loading Method and Evaluation of Efficiency

Cisplatin-loaded FA-2-DG was prepared by adding cisplatin into an aqueous solution of FA-2-DG at different concentrations. The mixture was stirred at 25 °C for 6 h in the dark, and the solution was then lyophilized to obtain the product. Fourier transform ion cyclotron resonance MS (solarix, Bruker Daltonics, Bremen, Germany) using electrospray ionization was used to verify the precise molecular mass of the compound [44]. Mass spectrometry coupled with HPLC (1260, Agilent, Santa Clara, CA, USA) was used to develop a method for its quantitative analysis. A hydrophilic interaction chromatography (HILIC) column (2.1 × 100 mm, 1.7 μm; Waters, Milford, MA, USA) was used for the separation. The mobile phase was water (A) and acetonitrile (B), and the gradient elution method was used: initially 5% A, increased to 40% in 50 min. The flow rate was 0.3 mL/min and the column temperature was 25 °C.

## 4. Conclusions

In this study, 2-DG-modified folic acid (FA-2-DG) was evaluated with optical characterization methods and morphological characterization. Its self-assembly was confirmed, and its formation of nanoparticles was verified. The water solubility of FA-2-DG was much greater than that of folic acid, which may explain these results. FA-2-DG also loaded cisplatin with coordination bonding, and the loading efficiency was maximal at a molar ratio of 2:1.
